# High-throughput terahertz imaging: progress and challenges

**DOI:** 10.1038/s41377-023-01278-0

**Published:** 2023-09-15

**Authors:** Xurong Li, Jingxi Li, Yuhang Li, Aydogan Ozcan, Mona Jarrahi

**Affiliations:** 1 Department of Electrical & Computer Engineering, University of California Los Angeles (UCLA), Los Angeles, CA 90095 USA; 2 California NanoSystems Institute (CNSI), University of California Los Angeles (UCLA), Los Angeles, CA 90095 USA; 3 Department of Bioengineering, University of California Los Angeles (UCLA), Los Angeles, CA 90095 USA

**Keywords:** Imaging and sensing, Terahertz optics, Optoelectronic devices and components

## Abstract

Many exciting terahertz imaging applications, such as non-destructive evaluation, biomedical diagnosis, and security screening, have been historically limited in practical usage due to the raster-scanning requirement of imaging systems, which impose very low imaging speeds. However, recent advancements in terahertz imaging systems have greatly increased the imaging throughput and brought the promising potential of terahertz radiation from research laboratories closer to real-world applications. Here, we review the development of terahertz imaging technologies from both hardware and computational imaging perspectives. We introduce and compare different types of hardware enabling frequency-domain and time-domain imaging using various thermal, photon, and field image sensor arrays. We discuss how different imaging hardware and computational imaging algorithms provide opportunities for capturing time-of-flight, spectroscopic, phase, and intensity image data at high throughputs. Furthermore, the new prospects and challenges for the development of future high-throughput terahertz imaging systems are briefly introduced.

## Introduction

Lying between the infrared and millimeter wave regimes, the terahertz frequency range is often referred to as the “terahertz gap”, because of the lack of efficient terahertz emitters and detectors. However, this gap is being gradually filled from both sides of the spectrum, as terahertz technology is developing in tandem with the more mature radio frequency (RF) and photonics industries^1,2^. The unique properties of terahertz waves have prompted numerous compelling applications. Higher carrier frequencies compared to millimeter waves promise unprecedented channel capacities, making terahertz waves serious candidates for signal carriers in 6 G and beyond wireless communication systems^3^. As the host of spectral signatures of many molecules, the terahertz spectrum has a wide range of applications in chemical identification, material characterization, atmospheric/astrophysics studies, and gas sensing^4–6^. The higher penetration through many non-conducting materials compared to infrared waves and shorter wavelength compared to millimeter waves, combined with the non-ionizing nature of the radiation, make terahertz waves excellent means for non-destructive testing, security screening, biomedical imaging, and cultural heritage conservation^7–10^.

Imaging systems operating at terahertz frequencies share some similarities but also distinctions with those operating in the infrared and millimeter wave regimes. While infrared image sensors generally rely on thermal^11^ and photon^12^ detection, field detection is the most dominant mechanism in millimeter-wave image sensors^13^. Terahertz imaging can be performed through all three mechanisms, i.e., thermal, photon, and field detection. Regardless, either the single-frequency or frequency-averaged response of the imaged object is captured through a frequency-domain terahertz imaging system, or the ultrafast temporal response of the imaged object in response to a pulsed terahertz illumination is captured through a time-domain terahertz imaging system. While most utilized terahertz image sensors are in a single-pixel format, this has not hampered the exploration of many exciting applications. For example, histopathological examination of basal cell carcinoma and melanoma specimens^14^ and coating thickness monitoring of pharmaceutical tablet^15^ were performed with terahertz time-domain sensors; ancient paintings^16^ and concealed suspicious objects^17^ were non-destructively examined by terahertz radars. However, the total imaging time for the above-mentioned applications ranges from tens of minutes to tens of hours due to the single-pixel nature of these imaging systems and the requirement for raster scanning to acquire the image data. To realize the full potential of terahertz imaging for real-world applications, the lengthy imaging process of traditional systems is gradually addressed by the development of terahertz image sensor arrays and advanced computational imaging algorithms.

In this article, we give an overview of the developments in high-throughput terahertz imaging systems. We introduce various image sensor arrays that have been utilized to develop terahertz imaging systems that support high-throughput operation. Then, we discuss approaches to modify the terahertz imaging hardware to enhance the imaging speed while trading off other imaging specifications. Next, we review various computational imaging methods that provide additional imaging functionalities and ease the restrictions of the imaging hardware to enable high-throughput terahertz imaging. Finally, we will summarize the high-throughput terahertz imaging techniques and share our thoughts on the challenges and opportunities for further advancements.

## Terahertz imaging systems based on image sensor arrays

Since the first demonstration of terahertz imaging in 1976^18^, numerous image sensors have been invented for terahertz imaging. However, not all types of image sensors are scalable to large arrays, which is a crucial requirement for high-throughput imaging. This section highlights high-throughput terahertz imaging systems based on various image sensor arrays. The performance of these terahertz imaging systems is quantified by their space-bandwidth product, sensitivity, dynamic range, and imaging speed within their operation frequency range. Space-bandwidth product is defined as the number of resolvable pixels in a captured image, where the minimum resolvable pixel size is determined by the diffraction limit or the physical pixel size of the image sensor, whichever is smaller. While the sensitivity of time-domain imaging systems is generally specified by the signal-to-noise ratio (SNR) of their image sensors, the sensitivity of frequency-domain imaging systems is usually quantified using the noise equivalent power (NEP) of the utilized image sensor, defined as the minimum detectable power per square root bandwidth that results in an SNR of unity. Dynamic range, defined as the ratio between the maximum and minimum detectable signals by the image sensor, determines the maximum achievable contrast in a resolved image. Generally, there is a tradeoff between sensitivity/dynamic range and imaging speed: increasing the integration time, in many cases, enhances the sensitivity/dynamic range at the expense of a reduced imaging frame rate.

### Frequency-domain terahertz imaging systems

**Fig. 1:**
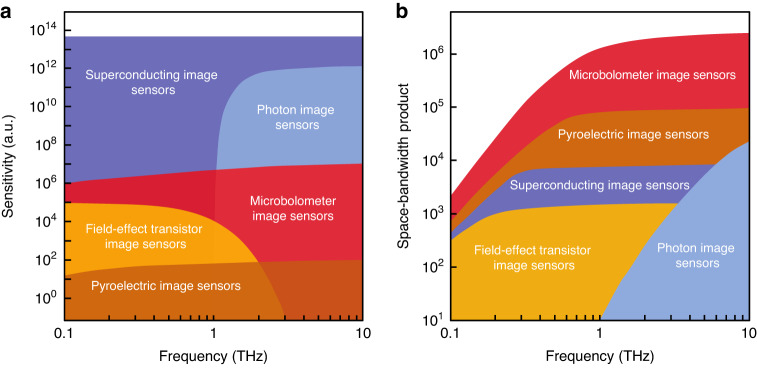
State-of-the-art frequency-domain terahertz image sensors. **a** Terahertz detection sensitivity as a function of frequency. **b** Space-bandwidth product as a function of frequency. Space-bandwidth product is defined as the product of the field of view and the spatial frequency range, representing the number of resolvable pixels in the image. At lower frequencies, the space-bandwidth product is determined by the diffraction limit, where the terahertz wavelength is larger than the pixel size. At higher frequencies, the space-bandwidth product is determined by the number of physical pixels

**Fig. 2:**
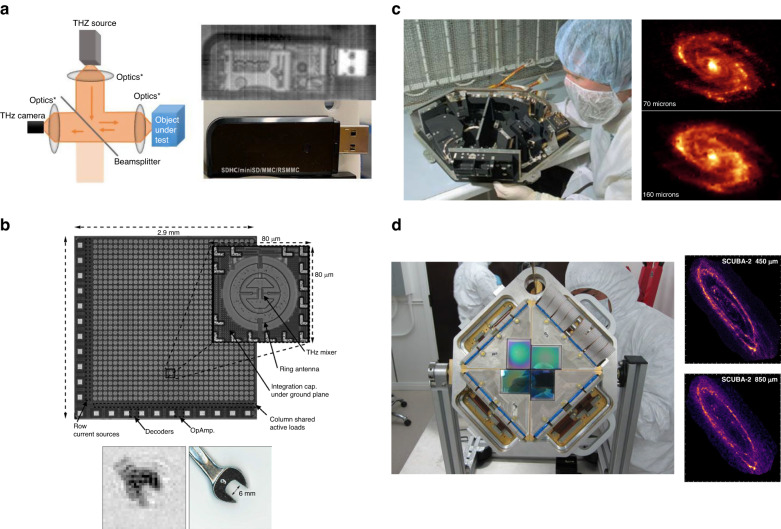
Example frequency-domain terahertz imaging systems based on image sensor arrays. **a** Terahertz imaging using a 384 × 288 VO_x_-based microbolometer image sensor^241^. An oscillator with two frequency doublers (manufactured by Virginia Diodes) is used as the terahertz source at 395 GHz. Left: Schematic diagram of the terahertz imaging setup. Right: The stitched terahertz image of a USB flash drive at 395 GHz. Each sub-image is captured with a 13.4 × 10.1 mm^2^ field of view at an imaging speed of 50 fps. **b** Terahertz imaging with a 32 × 32 FET image sensor array fabricated using a 65 nm CMOS technology^32^. A ×48 multiplier chain is used as the radiation source at 650 GHz, providing a 1.2 mW radiation power. Bottom: One image frame of a 6 mm wrench taken from a 25-fps video at 650 GHz. **c** Passive terahertz imaging with a photon image sensor array. Left: Photograph of the Multiband Imaging Photometer for Spitzer (MIPS) containing two Ge:Ga photoconductor arrays: an unstressed 32 × 32 array operating in the 50–100 µm wavelength range and a stressed 2 × 20 array operating at 160 µm wavelength^242^. Right: Images of the Messier 81 galaxy at 70 µm (top) and 160 µm (bottom)^243^. **d** Passive terahertz imaging with a superconducting image sensor array. Left: Photograph of one of the two focal-plane units on Submillimeter Common-User Bolometer Array 2 (SCUBA-2) for James Clerk Maxwell Telescope (JCMT)^244^. Each focal-plane unit comprises a 5120-pixel TES image sensor array, working at 450 and 850 µm wavelengths, respectively^42^. Right: Images of the Andromeda Galaxy at 450 µm (top) and 850 µm (bottom)^245^. **a** adapted with permission from ref. ^241^© 2020 SPIE. **b** adapted with permission from ref. ^32^ © 2012 IEEE. **c** reproduced courtesy of NASA/JPL-Caltech. **d** reproduced courtesy of NIST and the HASHTAG team

In the context of thermal terahertz imagers, microbolometers are one of the most widely used image sensors, which translate the temperature change caused by the received terahertz radiation into conductivity change in a thermistor material. Vanadium oxide (VO_x_) and amorphous silicon (α-Si) are the most popular thermistor materials used in room-temperature microbolometers. The architecture of these microbolometers is compatible with flip-chip mounted readout integrated circuits, facilitating the realization of large arrays. As a result, several terahertz imagers based on VO_x_ and α-Si microbolometer arrays have been commercialized^19–21^. Despite the trade-off between noise performance and response time, state-of-the-art room-temperature microbolometers can provide sensitivities up to 10^13^ √Hz/W (NEP levels down to 10^-13^ √Hz/W), space-bandwidth product as high as 1 million, and video-rate imaging speeds (Fig. 1)^20–22^. An example terahertz image captured using a microbolometer image sensor array is shown in Fig. 2a. For applications requiring higher sensitivity levels (e.g., astrophysics observations), microbolometers can be cryogenically cooled. For example, the Photodetector Array Camera and Spectrometer (PACS) bolometers, developed for Herschel Space Observatory, have a sensitivity close to the cosmic background^23^. However, their extremely low working temperature (0.3 K) imposes strong limitations on the system’s cost and size. In addition, restrictions of special readout circuits operating at such low temperatures have limited the space-bandwidth product of these cryogenic-cooled microbolometers compared to their room-temperature counterparts. Pyroelectric detectors are another category of thermal image sensors, which translate the temperature change caused by the received terahertz radiation into polarization change in pyroelectric crystals that can be sensed electronically. Due to the very broadband nature of the pyroelectric effect (1 μm < λ < 3000 µm), many pyroelectric cameras developed for infrared imaging are also used for video-rate terahertz imaging at room temperature^24^. However, they have lower sensitivity (sensitivity < 10^8^ √Hz/W, or NEP > 10^-8 ^W/√Hz) compared to other types of terahertz imagers. The temperature change caused by the received terahertz radiation can be also used for mechanical reshaping of meta-molecule optical reflectors, enabling the use of a visible camera for high-throughput terahertz imaging with a large number of pixels^25^.

For room-temperature terahertz imaging, field-effect-transistor (FET) image sensors are the main competitors to microbolometer image sensors. The operation of FET image sensors relies on the excitation of plasma waves inside the transistor channel, which induces a constant voltage across the transistor junctions through a nonlinear rectification process. Since the excitation of plasma waves is independent of the transistor parasitics, FET image sensors can detect terahertz electric fields even at frequencies higher than the transistor cutoff frequency. The physical principles of these image sensors were initially proposed by Dyakonov and Shur^26^, and experimentally demonstrated first with III–V FETs^27,28^ and later with Si FETs^29^. One of the main advantages of FET imager sensors is their excellent scalability. Especially, Si-FET image sensors are compatible with standard complementary metal oxide semiconductor (CMOS) processes and can be realized in large arrays integrated with the readout electronics. Thanks to their cost-effective and compact attributes, a number of terahertz imagers based on III–V and Si FETs are now commercialized^30,31^. Compared to room-temperature microbolometer imager sensors, FET image sensors usually work at lower terahertz frequencies and offer lower sensitivities (sensitivities below 10^11^ √Hz/W, or NEP levels higher than 10^-11 ^W/√Hz)^32–35^. However, since they do not use a thermal detection process, higher imaging speeds can be offered by FET image sensors. An example terahertz image captured using a FET image sensor array is shown in Fig. 2b.

As the most dominant image sensors in visible imagers, photon detectors also play a crucial role in terahertz imaging. Because of the low energy of terahertz photons, very few intrinsic semiconductors have small enough bandgap energies for terahertz photon detection. Instead, extrinsic semiconductors, quantum wells, and quantum dots are used to introduce interband and intersubband energy level separations smaller than terahertz photon energies. To prevent thermal-noise-induced carrier excitation, this type of terahertz image sensors should operate at cryogenic temperatures (typically <5 K), while offering very high sensitivities (sensitivity ~10^17^ √Hz/W, or NEP ~ 10^−^^17 ^W/√Hz)^23,36^. Apart from the cryogenic cooling requirement, there are two other limitations associated with terahertz photon detectors: operation frequency and scalability restrictions. The lowest demonstrated operation frequency of photon detectors has been ~1.5 THz with a stressed gallium-doped germanium substrate used as the extrinsic photon absorber^37^. Furthermore, terahertz photon detectors cannot be easily integrated with readout electronics because the thermal emission from the electronic circuits can disrupt the photon detection operation^38^. As a result, the demonstrated terahertz imagers based on photon detector image sensors have been assembled from small detection and readout units with limited space-bandwidth product^23^. A layer-hybrid readout architecture can potentially block the thermal emission from the readout electronics and enable larger pixel-count terahertz imagers^38,39^. An example terahertz image captured using a photon image sensor array is shown in Fig. 2c. Alternatively, the received terahertz photons from the imaged object can be converted to visible photons using quantum dots^40^ or laser-excited atomic vapors^41^ and an optical camera can be used for high-throughput imaging with a large number of pixels at room temperature. However, these THz-to-visible photon conversion processes require complex and bulky setups.

Superconducting terahertz imagers can provide similar or even better sensitivity compared to photon imagers. Meanwhile, they have better scalability and can work at the lower portion of the terahertz frequency band. There are four major types of superconducting imagers based on: transition edge sensors (TESs), kinetic inductance detectors (KIDs), kinetic inductance bolometers (KIBs) and quantum capacitance detectors (QCDs). TES image sensors work at the superconducting transition temperature and sense the temperature-dependent DC resistance at the onset of superconductivity. The largest pixel-count TES imager that has been demonstrated contains 5120 pixels and offers an NEP of 10^-16 ^W/√Hz (sensitivity of 10^16^ √Hz/W) at 0.1 K^42^. To support fast time-multiplexed readout from a large number of pixels, it uses a superconducting quantum interference device enabling imaging speeds as high as 180 fps^42^. KID and KIB image sensors measure the kinetic inductance variations in response to the received radiation. The absorption of terahertz photons breaks Cooper pairs, reducing their density and increasing the kinetic inductance^43^. KID imagers work at sub-Kelvin temperatures and can provide NEP values down to ~10^-19 ^W/√Hz (sensitivities up to 10^19^ √Hz/W)^44–47^. In contrast to KIDs, KIBs utilize the kinetic inductance’s temperature dependency and, therefore, relax the working temperatures to 5–10 K^48–50^. The largest pixel-count KIB imager demonstrated has 8712 pixels and offers an NEP of 10^−^^14 ^W/√Hz (sensitivity of 10^14^ √Hz/W)^51^. The operation of QCD image sensors also relies on Cooper pair breaking caused by the absorbed terahertz photons. The generated quasiparticles tunneling in or out of a superconducting island change the effective capacitance, which can be electronically measured. QCD imagers have provided the highest sensitivity among all demonstrated terahertz imagers with NEP levels as low as 10^−^^20 ^W/√Hz (sensitivities up to 10^20^ √Hz/W)^52,53^. The high sensitivity of terahertz image sensors based on superconductor and photon detectors enables fully staring passive imaging, while requiring operation at cryogenic temperatures^51^. Different from the time-multiplexing readout scheme used in TES imagers, KID, KIB, and QCD imagers adopt a frequency-multiplexing readout scheme, where a common feedline connects all the pixels and greatly reduces the complexity. An example terahertz image captured using a superconducting image sensor array is shown in Fig. 2d.

The frequency-domain terahertz imagers that have been discussed so far are capable of incoherent imaging and only resolve the intensity response of the imaged object. Coherent terahertz imaging can be realized using a heterodyne detection scheme to resolve both the amplitude and phase response of the imaged object^54^. By mixing the received radiation from the imaged object with a local oscillator (LO) beam and down-converting the terahertz frequency to an RF intermediate frequency (IF), high-performance RF electronics can be used for coherent signal detection. Superconductor-insulator-superconductor (SIS), hot-electron bolometer (HEB), Schottky diode, FET mixers, and photomixers can be used for THz-to-RF frequency down-conversion^55–58^. SIS and HEB mixers offer quantum-level sensitivities, however, they require cryogenic cooling to temperatures down to mK. Schottky diode and FET mixers operate at room temperature while offering lower sensitivity. Due to the complexity of the heterodyne detection architecture, the demonstrated coherent terahertz imagers have been limited to tens of pixels^59,60^. The largest demonstrated array has been a 64-pixel 0.34 THz imager based on cryogenically-cooled SIS mixers developed for astrophysics observations at the Heinrich Hertz Telescope^61^. For room-temperature coherent imaging, a 32-pixel 0.24-THz heterodyne array with integrated synchronized LOs has been demonstrated^62^.

### Time-domain terahertz imaging systems

**Fig. 3:**
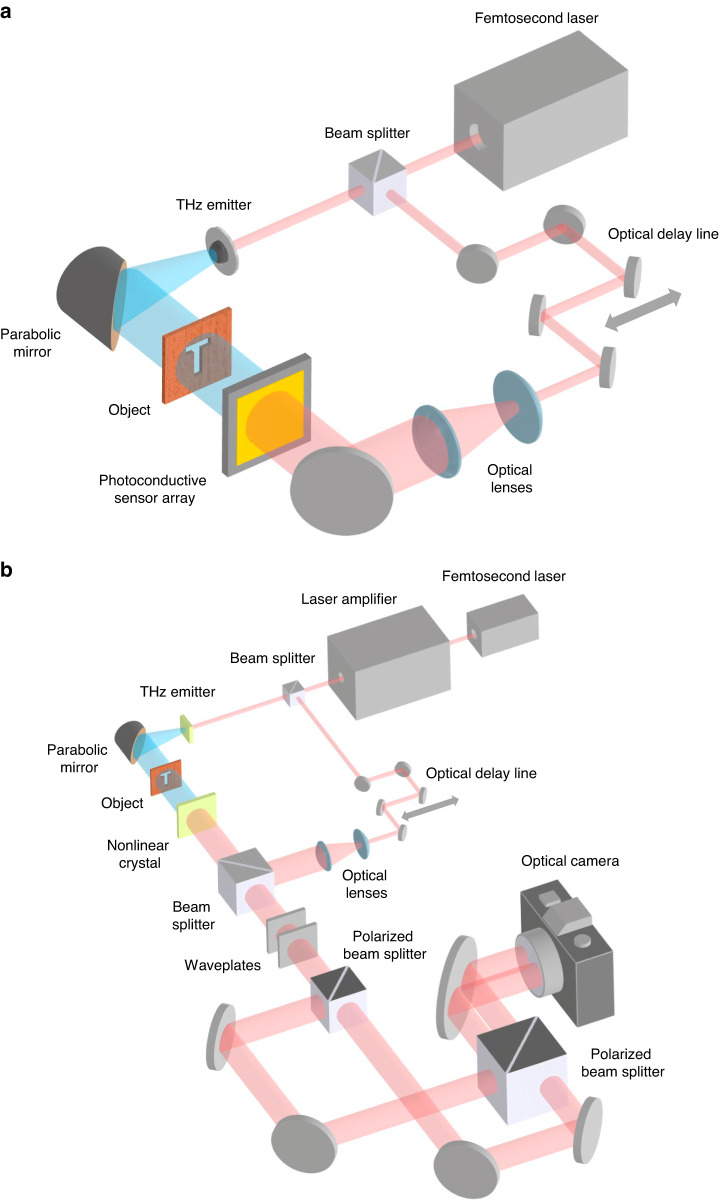
Terahertz time-domain imaging systems. **a** Photoconductive image sensor arrays, and **b**, electro-optic sampling using an optical camera

Terahertz pulsed imagers based on time-domain spectroscopy (TDS) form another type of coherent imager, which provide not only amplitude and phase, but also ultrafast temporal and spectral information of the imaged object. THz-TDS imaging systems use photoconductive antennas or nonlinear optical processes to generate and detect terahertz waves in a pump-probe imaging setup^63^ (Fig. 3). The optical beam from a femtosecond laser is split into pump and probe branches. A photoconductive antenna or a nonlinear optical crystal pumped by the femtosecond pump pulses generates terahertz pulses^64–67^, which illuminate the imaged object. The transmitted or reflected terahertz pulses, which carry the object information, are detected through photoconductive or electrooptic image sensors. When a terahertz pulse illuminates a photoconductive image sensor probed by a femtosecond optical pulse, the received terahertz field drifts photo-generated carriers and induces a photocurrent that is proportional to the instantaneous terahertz electric field^68–70^. By varying the time delay between the optical pump and probe beams, the time-domain electric field profile of the terahertz signal, which carries the ultrafast temporal information of the imaged object, is obtained. By taking the Fourier transform of the time-domain electric field, the terahertz radiation spectrum, which carries the spectral amplitude and phase information of the imaged object, is calculated. The time-domain electric field profile of the terahertz signal and the corresponding spectral information can also be obtained using electro-optic image sensors. The terahertz field detection mechanism in most electro-optic image sensors is the Pockels effect, where the received terahertz electric field changes the birefringence of a nonlinear crystal and hence the polarization of the optical probe beam propagating through the cystal^71–73^.

**Fig. 4:**
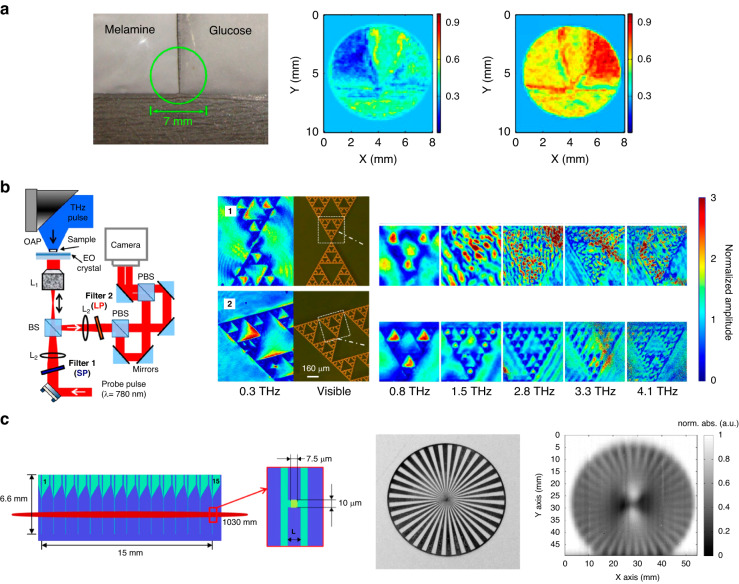
Example time-domain terahertz imaging systems based on electro-optic and photoconductive image sensor arrays. **a** Far-field terahertz time-domain imaging using electro-optic sampling with an optical camera. Left: Photograph of melamine and glucose powders, which have absorption peaks at 2.01 and 1.49 THz. Terahertz images can help identify melamine (center) and glucose (right)^78^. **b** Near-field terahertz time-domain imaging using electro-optic sampling with an optical camera. Left: The near-field imaging setup. Right: Terahertz near-field images of 100-nm-thick gold Sierpinski fractal antennas patterned on top of X-cut LiNbO_3_ crystals with thicknesses of 10 µm (b.1) and 1 µm (b.2)^92^. **c** Terahertz time-domain imaging with a 1D photoconductive antenna array. Left: The photoconductive antenna array with 15 dipole antennas. Middle and right: Optical and terahertz images of a metallic Siemen star, respectively^100^. **a** adapted with permission from ref. ^78^ © 2010 Elsevier B.V. All rights reserved.. **b** adapted with permission from ref. ^92^ © 2016 Optica Publishing Group. **c** adapted with permission from ref. ^100^ © 2014 Optica Publishing Group

While conventional THz-TDS imaging systems are typically single-pixel and require raster scanning to acquire the image data, arrays of electro-optic and photoconductive image sensors have been utilized to address the slow imaging speed and bulky/complex nature of single-pixel THz-TDS imaging systems. Since terahertz field detection in an electro-optic image sensor involves the detection of the optical probe beam, scaling a single-pixel electro-optic image sensor to an array is straightforward. An optical camera can capture the 2D profile of the optical probe beam after interaction with the terahertz beam in the electro-optic crystal, during which the object’s amplitude and phase information is converted from the terahertz beam to the probe beam. The first raster-scan-free THz-TDS imaging system based on electrooptic sampling was demonstrated using a ZnTe crystal and a CCD camera in 1996^74^. Despite the substantial increase in the imaging speed, the SNR and spectral bandwidth at each pixel are significantly lower compared to single-pixel THz-TDS imaging systems based on electro-optic sampling. This significant SNR and bandwidth reduction is due to the exposure of the electro-optic crystal to an unfocused terahertz beam to maintain the spatial information of the imaged object. The dramatic drop in the terahertz field intensity reduces the birefringence in the electro-optic crystal and the corresponding signal at each pixel. Since the first demonstration, many techniques were explored to address the SNR and bandwidth reduction problems, including the use of regenerative amplified lasers^75^, dynamic background subtraction^76^, lock-in detection in a time-of-flight camera^77^, and balanced electro-optic detection^78^. In order to provide sufficient birefringence in the electro-optic crystal to achieve acceptable SNR levels, they generally require high-energy amplified laser systems that provide μJ - mJ optical pulse energies^78–83^. An example terahertz image captured using electro-optic sampling with an optical camera is shown in Fig. 4a.

Raster-scan-free THz-TDS imaging systems based on electro-optic sampling can also be used for near-field terahertz imaging. The working principles are the same as far-field imaging, except that the imaged object is in direct contact with the nonlinear crystal^84–93^. Interaction between the near-field terahertz response of the imaged object and the optical probe beam in the nonlinear crystal, enables resolving much higher resolution images beyond the diffraction limit. While higher imaging resolution can be achieved using thinner electro-optic crystals^94^, the shorter interaction length between the terahertz and optical probe beam reduces the terahertz field detection sensitivity. Therefore, the thickness of the electro-optic crystal should be carefully chosen in these near-field imaging systems while considering the tradeoff between image resolution and SNR. By eliminating the scanning probes or apertures used in other near-field imaging techniques, much faster image acquisition speeds can be achieved^95^. The first raster-scan-free, near-field, electro-optic terahertz imaging system was demonstrated in 2011^89^. Using a 1 µm-thick LiNbO_3_ crystal, a spatial resolution of 5 μm (λ/600 at 100 GHz) was achieved^92^ (Fig. 4b). In the following developments, the use of intense terahertz pulse sources with electric fields exceeding hundreds of kV/cm^94^ and alternative nonlinear crystals with the Kerr nonlinearity effect instead of the Pockels effect^96^ offered enhanced image contrast and resolution. Consequently, free induction decay signals from a tyrosine crystal^88^ and laterally propagating electric field around split-ring resonators^91,97^ were observed using this near-field terahertz imaging scheme.

Another direction for raster-scan-free THz-TDS imaging is using an array of photoconductive image sensors. Early work explored the use of 1D photoconductive antenna arrays with the data readout performed by multi-channel lock-in amplifiers, offering up to a 39 dB SNR and spectral bandwidth of 0.8 THz^98–100^ (Fig. 4c). However, allocating a lock-in amplifier channel to each pixel is not a scalable readout architecture, limiting the total number of pixels of the demonstrated 1D photoconductive antenna arrays to 16. While using 1D photoconductive antenna arrays enables concurrent acquisition of the image data along one axis, raster scanning along the other axis is still required to capture the 2D image data, limiting the imaging speed. Another major challenge in developing high-efficiency photoconductive image sensors is the fundamental limit on the optical fill factor associated with the discrete architecture of conventional photoconductive antennas. Conventional photoconductive image sensors use discrete terahertz antennas connected to optically-probed photoconductive active areas much smaller than the terahertz antenna area. Therefore, an array of image sensors comprised of conventional photoconductive antennas has a very low fill factor, which results in very poor optical probing efficiency. To address this limitation, a plasmonic photoconductive terahertz focal-plane array (THz-FPA) was recently developed for raster-scan-free THz-TDS imaging^101^. By increasing the optical fill factor and maximizing the spatial overlap between the photocarriers and terahertz electric field, SNR levels as high as 81.0 dB and spectral bandwidths exceeding 4 THz were achieved without a multi-channel lock-in amplifier. Using a multiplexing electrical readout, a time-domain imaging speed of 16 fps was achieved, enabling the capture of the terahertz time-domain video of water flow^101^. Furthermore, the multispectral amplitude and phase data provided by this THz-FPA was used to super-resolve both the shape and depth of 3D structures with a lateral/depth resolution as small as 60/10 μm and an effective number of pixels exceeding 1-kilo-pixels^101^.

Raster-scan-free THz-TDS imaging systems based on photoconductive and electro-optic image sensors enable concurrent data acquisition from all pixels. However, attributes of the optical delay stage required for time-domain scanning impose another limitation on the overall imaging speed. The conventional method to introduce variable optical delay is mounting a pair of mirrors on a linear mechanical motorized stage. Faster mechanical delay lines based on voice-coil-driven mirrors^102^, rotary mirrors^103^, and rotary dielectrics^104^ provide 10–100 traces per second with a temporal range of ~10 s ps. An acousto-optic delay can enable 36,000 traces per second in a 12.4-ps time window^105^. Another way to increase the speed of optical delay scanning is by non-mechanical time-domain sampling methods, such as asynchronous optical sampling (ASOPS)^106,107^ and electronically controlled optical sampling (ECOPS)^108,109^. These methods enable optical sampling speeds exceeding 100 kHz^107^ and a very large (for the case of ASOPS) or adjustable (for the case of ECOPS) time window, with the drawback of requiring two costly femtosecond lasers. Alternatively, optical sampling by cavity tuning (OSCAT)^110^, single-laser polarization-controlled optical sampling (SLAPCOPS)^111^, and single-laser self-triggered ASOPS^112^ utilize only one femtosecond laser for optical sampling with the drawback of a slower time-domain scanning speed. It should be noted that non-mechanical time-domain sampling methods are less compatible with electro-optic imagers, which usually use regenerative amplified lasers with high pulse energies and kHz-range repetition rates.

### Functionalities and limitations of terahertz imaging systems based on image sensor arrays

**Fig. 5:**
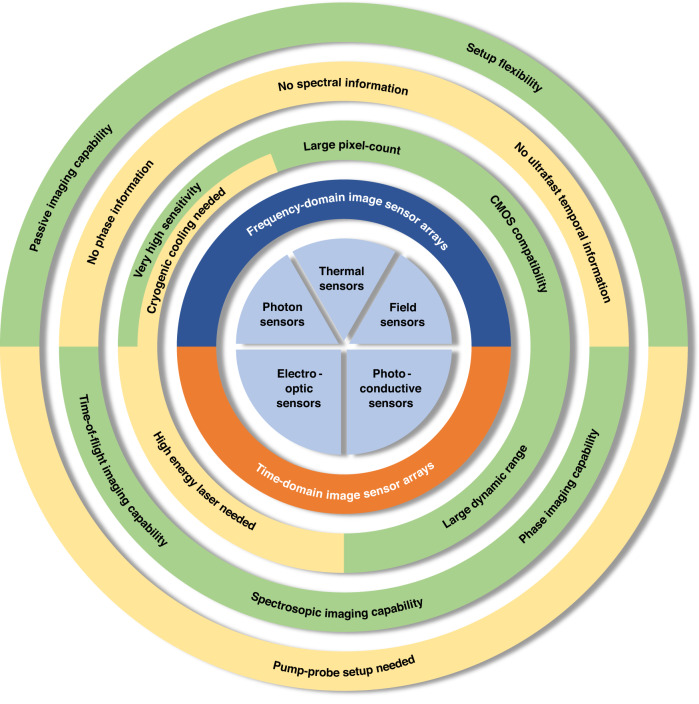
Summary of functionalities and limitations of different terahertz imaging systems based on image sensor arrays. Tradeoffs between imaging systems based on frequency-domain and time-domain image sensor arrays

Figure 5 highlights the functionalities and limitations of different terahertz imaging systems based on image sensor arrays. Frequency-domain imaging systems only resolve the amplitude response of the imaged object at a single frequency or a broad range of frequencies, without obtaining the ultrafast temporal and multispectral information. In the meantime, they have flexible setups that can be used for both passive and active terahertz imaging using different types of terahertz illumination sources. Time-domain imaging systems resolve both the amplitude and phase response of the imaged object, as well as the ultrafast temporal and multispectral information. However, they can only be used for active terahertz imaging and require a pump-probe imaging setup with a variable optical delay line, increasing the size, cost, and complexity of the imaging hardware.

**Fig. 6:**
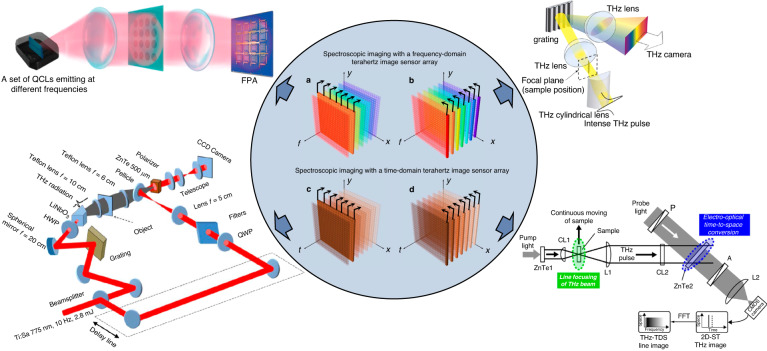
Various schemes for spectroscopic terahertz imaging. **a** Using a 2D image sensor array in a frequency-domain imaging system, the complete spectroscopic dataset $I(x,y,f)$ can be constructed by scanning the illumination frequency and recording the 2D image data at each illumination frequency^114^. **b** The frequency-domain imaging system with a 2D image sensor array can be converted to a 1D spectroscopic image sensor array using dispersive terahertz optics, and the complete spectroscopic data cube can be constructed by spatial scanning along the other dimension^119^. **c** In conventional terahertz time-domain imaging systems, the spectroscopic dataset $I(x,y,f)$ is obtained by taking the Fourier transform of the time-domain image data $I(x,y,t)$, where the optical delay between the pump and probe beams is varied and the 2D image data is captured at each temporal point^82^. **d** Alternatively, the time-domain information can be encoded in one dimension of the image sensor array, while using the other dimension for recording the spatial information. The entire time-domain data cube, ${I}(x,y,t)$, can be constructed with 1D spatial scanning^123^. **a** adapted with permission from ref. ^114^ © 2018 the Authors under the terms of CC-BY. **b** adapted with permission from ref. ^119^ © 2017 Natsuki Kanda et al under the terms of CC-BY 4.0. **c** adapted with permission from ref. ^82^ © 2018 Optica Publishing Group. **d** adapted with permission from ref. ^123^ © 2010 Optica Publishing Group

While the functionalities of terahertz imaging systems are generally determined by the above-mentioned principles, it is possible to make modifications in their operation framework to achieve new and/or enhanced capabilities. This is accompanied by possible degradation in other imaging specifications, e.g., speed, SNR, or system complexity. For example, spectroscopic imaging with a frequency-domain terahertz imager can be performed by frequency scanning through switching among different terahertz sources^113,114^, tuning the frequency of a single source^115,116^, or applying different terahertz filters^117,118^ at the expense of much lower imaging speed and higher setup complexity. This modified operation framework is illustrated in Fig. 6a, where a complete spectroscopic data set $I(x,y,f)$ is depicted by a 3D datacube in the ${xyf}$ space (${xy}$: the 2D spatial plane, $f$: the frequency axis). For a frequency-domain imager, the captured image data at a given illumination frequency ${f}_{0}$ is $I(x,y,{f}_{0})$, a 2D slide of the datacube. By scanning the illumination frequency and recording the 2D slides of the datacube at different frequencies, the complete spectroscopic image data $I(x,y,f)$ is collected. An alternative approach to performing spectroscopic imaging with a frequency-domain terahertz imager is to incorporate dispersive terahertz optics and use the imager as a line-scanning spectrometer^119,120^. The dispersive optics maps different frequency components onto 1D positions on the imager, where the other dimension can still record the spatial image information. With an additional 1D spatial scanning, the complete spectroscopic image data can be obtained (Fig. 6b) at the expense of much lower imaging speed and higher setup complexity. Such a line-scanning spectroscopic imaging approach would be attractive when the movement of imaging objects is automated in one dimension (e.g., on a conveyor). Through this approach, spectroscopic imaging within the bandwidth of 1.3 – 2.1 THz was performed with a frequency resolution ranging from 30 to 70 GHz and a 1D imaging speed of 15 fps using a microbolometer array^119^.

A similar tradeoff exists for spectroscopic imaging with a time-domain terahertz imager. Conventional THz-TDS imaging systems use a variable optical delay between the pump and probe beams to acquire the 2D image data at different temporal points (Fig. 6c). The complete spectroscopic image data $I(x,y,f)$ is obtained by taking the Fourier transform of the time-domain image data $I(x,y,t)$. An alternative method to realize spectroscopic imaging with a THz-TDS imager is to encode the time-domain information in one dimension of the image sensor array, while the other dimension can still record the spatial information. With an additional 1D spatial scanning, the complete $I(x,y,t)$ datacube can be obtained (Fig. 6d). This can be achieved through non-collinear electro-optical time-to-space conversion by tilting the wave front of the terahertz radiation^121–125^, the optical probe beam^126,127^, or both^128^. This approach is specifically advantageous when the movement of the imaged object is automated in one dimension, and is accompanied by a degradation in the temporal range and frequency resolution.

Another example of a modified terahertz imaging framework is phase imaging with a frequency-domain terahertz imager through digital holography methods at the cost of system complexity. A coherent terahertz beam is separated into two paths: one impinging on the imaged object (object beam), and the other serving as a reference (reference beam). Interferograms of the two beams are recorded by the terahertz image sensor array and digitized. With the prior knowledge of the reference beam, the object beam at the image plane is calculated, and the complex object information is reconstructed by back-propagating the object beam from the imager plane to the object plane. More details on computational terahertz holography systems are provided in the next section.

## Computational terahertz imaging

As discussed in the previous sections, image sensor arrays have been instrumental in realizing high-throughput terahertz imaging. Operation principles, specifications, and limitations of different types of terahertz image sensor arrays used in both frequency-domain and time-domain systems were discussed. This section introduces various computational imaging methods that provide additional imaging functionalities and ease the restrictions of terahertz image sensors for high-throughput operation.

### Computational terahertz holography

Holography methods allow extracting the object information from the interferograms of two beams interacting with the object and a reference. Terahertz holography systems utilize off-axis or in-line interference. In in-line digital holography, object and reference beams travel along the same direction when recorded by the terahertz image sensor array. To minimize the distortion of the reference beam, the object must be fairly transparent or smaller than the terahertz beam. In-line digital holography requires additional effort to separate the interferograms from autocorrelation components, as well as the real image from the virtual image conjugate^129,130^. In off-axis digital holography, an angle is introduced between the object and reference beams to help separate interferograms from other autocorrelation components in the spatial frequency domain. Since the first demonstrations of digital holography with 2D terahertz image sensors^131,132^, terahertz digital holography has evolved rapidly with both hardware and algorithm improvements. Dual-frequency reconstruction was utilized to overcome the 2π phase ambiguity when unwrapping the phase response of thick objects^133^. Image quality and resolution were improved by recording holograms at multiple imaging planes to suppress image artifacts^134,135^, lateral shifting of the image sensor array to synthesize a larger detecting area^136,137^, and sub-pixel shifting of the image sensor array or the object to enable pixel super-resolution^129,135^. Today’s terahertz digital holography systems can offer video-rate (50 fps) imaging speeds^138^ and lateral resolution down to 35 μm (${\sim}\! {\lambda} /3$ at 2.52 THz)^130^. Compared to phase imaging through THz-TDS imaging systems, terahertz digital holography does not require femtosecond laser-based setups and is more cost-effective. The choice of terahertz source and image sensors array is more flexible and can be optimized according to the operation frequency. However, terahertz digital holography imposes more limitations on the imaged object and is restricted when imaging multi-layered and/or highly lossy objects^139^.

### Single-pixel terahertz imaging through spatial scene encoding

**Fig. 7:**
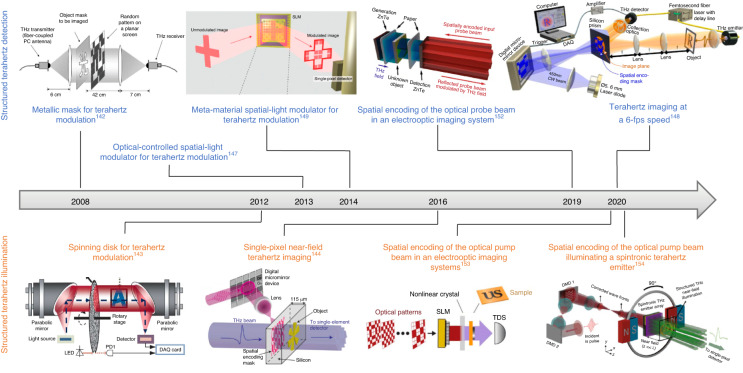
Advancements in single-pixel terahertz imaging systems using spatial beam encoding. Images adapted with permissions from ref. ^142^ © 2008 AIP Publishing, from ref. ^149^ © 2014, Springer Nature Limited, from © 2019 the Authors under the terms of CC-BY 4.0, from ref. ^148^ © 2020 the Authors under the terms of CC-BY 4.0, from ref. ^143^ © 2012 Optica Publishing Group, from ref. ^144^ © 2016 the Authors under the terms of CC-BY 4.0, from ref. ^153^ © 2020 the Authors under the terms of CC-BY, and from ref. ^154^ © 2020 the Authors under the terms of CC-BY 4.0

In contrast to the direct image capture with a terahertz image sensor array, a single-pixel terahertz image sensor can be used to reconstruct the image of an object by sequentially measuring/recording the terahertz response of the spatially modulated scene with a known spatial pattern sequence^140,141^. This imaging scheme benefits from the superior performance (e.g., SNR, dynamic range, operation bandwidth) of most single-pixel terahertz image sensors compared to terahertz image sensor arrays for both frequency-domain and time-domain imaging systems. Analogous to conventional raster-scanning-based imaging systems, the simplest scheme to resolve an *N*-pixel image through single-pixel imaging is to sequentially measure the terahertz response of the spatial region corresponding to each pixel, while blocking other pixels, with the total number of measurements, *M*, being equal to the number of pixels, *N*. Since the terahertz radiation corresponding to *N*-1 pixels is blocked in each measurement, this scheme has a low power efficiency. While random spatial modulation patterns can provide fairly good single-pixel image reconstruction results^142,143^, the use of orthogonal pattern sets, such as Hadamard^144,145^ and Fourier basis^146^, provides higher SNRs. Furthermore, since most natural images are sparse when represented in an appropriate basis, compressive sensing algorithms can reconstruct the image with fewer measurements than the number of pixels (*M* < *N*), enabling higher imaging speeds. The faster imaging operation is also supported by the fact that spatial modulation of terahertz response can be performed much faster than mechanical raster scanning using spinning disks^143^,optically-controlled spatial light modulators (SLMs)^147,148^, meta-material SLMs^149–151^, spatial encoding of the optical probe beam in electro-optic imaging systems^152^, and spatial encoding of the optical pump beam in imaging systems using nonlinear^153^ or spintronic terahertz emitters^154^. Single-pixel amplitude-only imaging^148^, broadband imaging^155^, and time-domain imaging^156^ have been realized in both far-field^143,148,149^ and near-field^154,157,158^ settings. Imaging speeds as high as 6 fps were demonstrated for amplitude-only imaging^148,159^. Figure 7 summarizes the development of single-pixel terahertz imaging systems. It should be mentioned that compressive sensing algorithms are not only applicable to single-pixel imaging and can also be used to increase the imaging throughput of multi-pixel image sensor arrays^160^.

### Computational terahertz imaging via diffractive processing

The terahertz imaging systems described so far follow a paradigm that relies primarily on computer-based digital processing to reconstruct the desired image. However, it is important to note that digital processing-based reconstruction is not without limitations. Due to the large amount of measurement data to be processed, a significant computational burden is imposed on the digital processing modules, resulting in substantial resource consumption and high output latency. Additionally, when the measurement data are inadequate or inaccurate, restoring lost information becomes challenging, even with robust algorithms and prior knowledge.

In the general discussion of computational optical imaging, the entire architecture of the imaging hardware can be analyzed as a combination of an optical encoder and an electronic decoder^161^. The front-end optical component can be seen as encoding the information of the object, while the back-end electronic part performs the decoding process. In the middle, the focal-plane sensor array plays a crucial role in the optoelectronic conversion process. Given the considerable design challenges posed by terahertz components and sensors, especially in aspects such as pixel count, dynamic range, and signal-to-noise ratio, one can consider the focal-plane array as an important bottleneck in the context of computational terahertz imaging. To address some of these challenges, an optimal strategy could be to engineer the optical front-end for task-specific optical encoding, and enable it to take over some of the computational tasks typically handled by the digital back-end.

In contrast to conventional optical devices employed for computational imaging within the visible spectrum, the design of a terahertz front-end exhibits unique characteristics. The use of planar diffractive optical elements (DOEs) is particularly advantageous in developing powerful terahertz front-ends, thanks to their inherent customizability and ease of fabrication due to the relatively large wavelength. For example, diffractive lenses can be engineered by manipulating their surface topology or the distribution of their refractive index. These components can be fabricated through 3D printing or laser cutting, forming components capable of modulating the amplitude and/or phase of the beam^162–165^. In terahertz imaging, diffractive lenses find their most widespread application as Fresnel lenses incorporated within raster scanning systems, primarily for reducing the size of the focused light spots. Additionally, some applications utilize multifocal diffractive lenses to achieve wide-field, broadband, or extended-depth-of-focus imaging^166–170^. Other diffractive lens designs offer capabilities of beam shaping^171^ and generation of intricate patterns, such as Airy^172,173^, Bessel^174–176^, and vortex beams^177,178^, expanding their utility in diverse terahertz imaging applications. Another possibility to precisely manipulate the phase distribution involves using metasurfaces based on the interaction of terahertz radiation with an array of resonators to form spatially varying phase changes. Some of the mainstream methods developed to introduce phase delay in dielectric metasurfaces include truncated waveguide^179,180^, geometrical phase^181,182^, and resonant/Huygens nanoantennas^183,184^. In recent years, much research has been dedicated to designing metasurface-based terahertz DOEs with properties such as broadband achromaticity, tunability, multi-foci and sub-diffraction characteristics^185–188^.

**Fig. 8:**
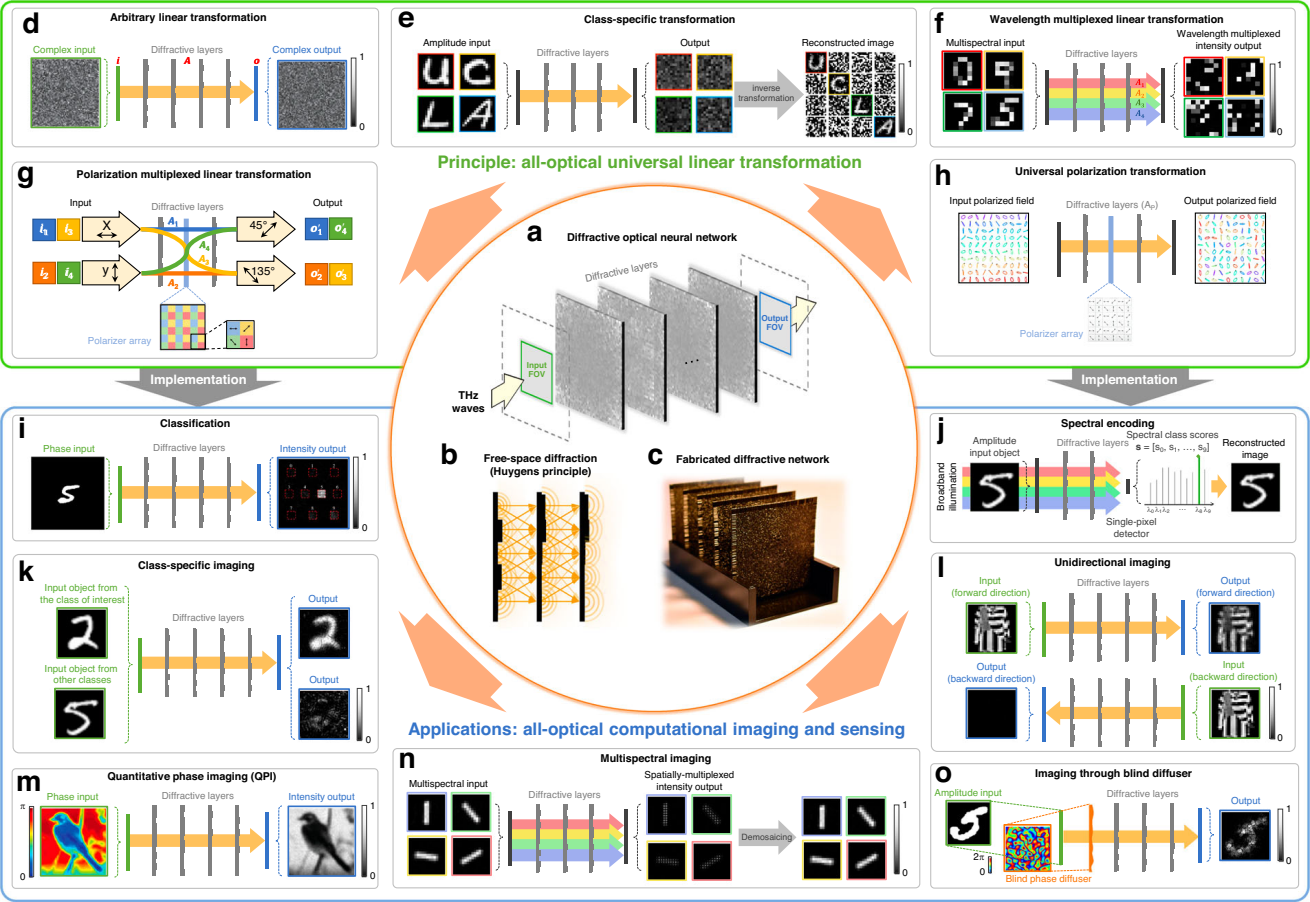
A schematic **summarizing** the computational terahertz imaging systems based on diffractive deep neural networks (D^2^NNs). **a** The schematic of a diffractive deep neural network (D^2^NNs). A D^2^NNs is composed of multiple transmissive (or reflective) layers, each comprising hundreds of thousands of diffractive neurons (features) with trainable transmission (or reflection) coefficient, which collectively modulate the incoming optical fields from an input FOV to produce desired field distributions within its output FOV. **b** The operation principle of a D^2^NNs is based on free-space diffraction of light (Huygens principle), where each diffractive neuron on a layer serves as a point source of secondary waves that connect to the neurons in the next layer. **c** A photograph of the fabricated diffractive network. **d**–**o** Different applications of D^2^NNs, including **d** all-optical universal linear transformation, **e** class-specific linear transformation, **f** wavelength-multiplexed linear transformations, **g** polarization-multiplexed linear transformations, **h** universal polarization transformation, **i** all-optical object classification, **j** spectral encoding and single-pixel object classification, **k** class-specific imaging, **l** unidirectional imaging, **m** quantitative phase imaging, **n** multispectral imaging, and **o** seeing unknown objects through a random diffuser. **e** adapted with permission from ref. ^200^ © 2023 the Authors under the terms of CC-BY 4.0. **f** adapted with permission from ref. ^210^ © 2023 SPIE. **g** adapted with permission from ref. ^214^ © 2022 the Authors under the terms of CC-BY 4.0. **j** adapted with permission from ref. ^212^ © 2021 the Authors under the terms of CC-BY 4.0. **k** adapted with permission from ref. ^209^ © 2022 the Authors under the terms of CC-BY. **l** adapted with permission from ref. ^208^ © 2023 AAAS. **m** adapted with permission from ref. ^204^ © 2023 John Wiley. **n** adapted with permission from ref. ^211^ © 2023 the Authors under the terms of CC-BY 4.0. **o** adapted with permission from ref. ^205^ © 2022 the Authors under the terms of CC-BY

Recently, a new optical information processing framework that incorporates multiple optimizable diffractive layers in a cascaded manner has also emerged, wherein these diffractive surfaces, once optimized, can collectively perform a complex function between the input and output fields-of-view using light-matter interactions, as shown in Fig. 8a. Referred to as a diffractive deep neural network (D^2^NN)^189,190^, this architecture is trained/optimized using deep learning methods in a data-driven fashion. Within this framework, the trainable variables are constituted by the complex-valued transmission coefficients of thousands of diffractive features distributed across each diffractive layer. Between different diffractive layers, the features are connected through the diffraction of light in free space, as illustrated in Fig. 8b. After the training process, the D^2^NN design can be physically fabricated to form an optical processing unit, as presented in Fig. 8c, which can all-optically perform transformations between its input and output field of views (FOVs) to achieve a specific computation or processing task, such as information encoding, classification, and detection. In contrast to conventional methods that rely on the combination of lens-based imaging using focal plane arrays or image sensors and digital processing of the sensor-captured signals, employing a diffractive optical front-end allows for the direct manipulation and processing of the input optical information in a highly parallel manner. This diffractive visual computing strategy provides an efficient and scalable alternative to the traditional machine vision pipeline undertaken by conventional optical imaging front-end and computational processing performed by a digital back-end. Moreover, since the D^2^NN framework solely employs passive optical elements, it requires no external energy for computing, except the illumination at the input. Compared to conventional optical processing platforms, this framework also possesses a compactness advantage, with the diffractive volume typically spanning only a few tens of wavelengths along the axial direction, showcasing an extremely low latency in addition to low-power consumption.

In essence, a D^2^NN can be considered a universal linear optical transformer, as depicted in Fig. 8d. It has been proven that the optical transformation embodied by a D^2^NN can approximate any arbitrarily-selected, complex-valued linear transformation, provided that the total number of trainable diffractive features in the diffractive network is no fewer than the degrees of freedom in the target linear transformation (i.e., the product of the degrees of freedom at the input FOV and the output FOV)^191,192^. Furthermore, a depth-related performance advantage is achieved by incorporating multiple, successive diffractive layers within a D^2^NN design. Both theoretical and numerical analyses presented in the literature^191,192^ reveal that, given a fixed number of diffractive features, allocating these features solely on one diffractive layer would result in significantly reduced performance instead of distributing them across two or more consecutively arranged diffractive layers. Given a specified target linear transformation, deeper D^2^NN designs can deliver superior transformation accuracy, diffraction efficiency, and optical signal contrast at their output.

D^2^NNs have been demonstrated with numerous applications in terahertz imaging and sensing tasks, particularly for performing task-specific statistical inference on input objects or scenes. A typical example involves all-optical object classification, whereby a detector array corresponding to the number of data classes is positioned at the output plane of a D^2^NN, and the classification result is determined by the maximum intensity measured by the output detectors, as illustrated in Fig. 8i. In this context, D^2^NNs were reported to classify amplitude-modulated MNIST handwritten digits, phase-modulated Fashion-MNIST product objects and grayscale CIFAR-10 image objects^189,190,193^. To experimentally demonstrate D^2^NN-based image classifiers, 3D printing was used to fabricate the diffractive layers of the trained network and assemble them into a physical neural network. A single-frequency continuous terahertz source operating at 400 GHz (~0.75 mm) was employed to illuminate 3D-printed objects (amplitude or phase), and a terahertz detector was scanned at the output plane to obtain the intensity measurements of the D^2^NN outputs. These measurements aligned well with numerical simulations and accurately identified the classes of the input objects. The optical classification accuracy of D^2^NNs can be enhanced with design improvements, including differential detection schemes^193^, time-lapse detection schemes^194^, class-specific^195^ and ensemble learning-based^195^ multi-D^2^NN parallel configurations, reaching, e.g., >98.5% and >62% for the classification of MNIST handwritten digit objects and CIFAR-10 images, respectively. These improvements, however, necessitate an increase in the number of output detectors or individual diffractive networks, the introduction of additional optical routing, or the measurement time.

Expanding on the capabilities of terahertz all-optical object classification using diffractive networks, D^2^NNs can also be coupled with electronic back-end processors or digital neural networks to improve their classification performance;^190^ when compared with traditional systems that combine terahertz cameras with electronic processors, this approach delegates part of the processing by the electronic processing system to the optical front-end, thereby showing advantages of enhanced inference speed and reduced need for high pixel count at the detector array. D^2^NNs have also been demonstrated with similar applications to perform statistical inference of input objects at other electromagnetic bands, such as visible light, near-infrared, and microwave. These applications encompass object classification^196–199^, image encryption^200^, image segmentation and saliency detection^201^, and human motion recognition^202,203^. Owing to the wavelength-dependent scalability of the free-space diffraction-based processing that D^2^NNs rely on, these unique designs, once trained using a certain spectral band, can be readily scaled physically to adapt to and operate at other parts of the electromagnetic spectrum.

The statistical inference tasks introduced above leverage D^2^NNs as all-optical encoders to extract and compress information from an input scene/FOV, outputting an optimized set of specific feature information. Another design paradigm involves using D^2^NNs to create imaging systems that preserve a high space-bandwidth product in their outputs. With their unique ability to perceive and process optical wavefronts, D^2^NN can undertake tasks beyond the capabilities of conventional lens-based imaging systems. A typical example of this is quantitative phase imaging (QPI), as illustrated in Fig. 8m^204^. In this case, D^2^NNs were trained to perform phase-to-intensity transformations from the input FOV to the output FOV in a snapshot, which enables the quantitative phase information of the input object to be obtained through the relative variations in optical intensity within the output FOV, serving as an all-optical substitute for digital phase-recovery algorithms. In another example, D^2^NNs were designed to enable imaging through scattering media, allowing all-optical reconstruction of unknown objects behind a random diffuser never seen during the training, as shown in Fig. 8o^205–207^. This demonstrates the generalization capability of this diffractive computational framework, revealing its robustness to unpredicted perturbations of the wavefront. Moreover, D^2^NNs can be trained to conduct unidirectional imaging tasks^208^. As shown in Fig. 8l, a D^2^NN facilitates the imaging of input objects only in the forward direction, while effectively blocking the imaging process in the reverse direction, thus breaking the conventional symmetry of lens-based imaging systems. Other applications of D^2^NNs in computational imaging include class-specific imaging and encoding of input objects^200,209^ (see Fig. 8e, k), which enhances imaging functionality with its statistical inference capacity. These applications have been experimentally validated in the terahertz band, with hardware system designs and implementations akin to those in prior examples^189^.

D^2^NNs can also access and handle other types of information present at the input FOV, such as the optical spectrum. By engineering the thickness profiles of diffractive layers based on their dispersion properties, one can design a broadband D^2^NN to form a wavelength-multiplexed linear transformation processor^210^, as illustrated in Fig. 8f. This processor can execute, in parallel, hundreds to thousands of distinct linear transformations across multiple wavelengths, given a proportionate increase in the number of trainable diffractive features according to the number of target operating wavelengths. Building on this principle, broadband D^2^NNs can be employed for multispectral imaging tasks^211^, as depicted in Fig. 8n. For example, researchers successfully designed a D^2^NN to function as a virtual filter array, which enables the imaging of an input object at up to 16 different target spectral bands (simultaneously), without using any additional spectral filters. The feasibility of this concept for terahertz multispectral imaging was experimentally confirmed in the same study. Furthermore, as illustrated in Fig. 8j, D^2^NNs can be designed as spatial-spectral encoders with statistical inference capabilities for spectral encoding and classification of objects^207,212^. Under pulsed illumination, D^2^NNs encode the spatial information of an object onto distinct wavelengths on a single-pixel spectroscopic detector, with the measured power intensity at each wavelength representing a probability score corresponding to an object data class. This feature facilitates all-optical object classification using a single pixel by selecting the wavelength with the maximum spectral class score at the output. The same research also presented that a shallow digital neural network-based decoder can be trained to reconstruct the input object image from each output spectral class score, thereby achieving spectral-based image encoding and decoding. Researchers verified these designs using a THz-TDS setup, successfully achieving high-accuracy classification and high structural-fidelity reconstruction of the input amplitude objects using a spectral band of ~200–300 GHz. Recently, similar concepts and methods have been used to illustrate the applicability of terahertz broadband D^2^NNs for rapid, non-destructive inspection of hidden defects in objects^213^. All these works collectively highlight the significant potential of D^2^NNs for developing various terahertz hyperspectral imaging and intelligent machine vision systems.

D^2^NNs can also be designed to harness the polarization information of light. As illustrated in Fig. 8h, by inserting multiple deterministic polarizer arrays into a trainable, isotropic D^2^NN architecture, a polarization-sensitive optical processing unit can be realized to process polarized input optical fields^214,215^. Analyses have shown that, by using different combinations of polarization states at the input and output FOVs, a polarization-encoded D^2^NN can implement up to four different independent linear transformations, albeit at the expense of requiring a four-fold increased number of diffractive features compared to a standard isotropic design;^214,215^ see Fig. 8g. By combining the polarization manipulation capabilities of D^2^NNs with spatial and spectral encoding, there is promising potential for developing terahertz camera systems with polarization-aware information processing capabilities, which could be instrumental in advanced terahertz polarization imaging and sensing instrumentation.

It is worth noting that the various terahertz implementations of the aforementioned D^2^NN designs often confront practical challenges arising from fabrication errors, misalignments between components, and other error factors, leading to a degradation in the performance of the experimental systems. However, these issues can be mitigated by “vaccination” strategies^216,217^. Specifically, the 3D printing or fabrication errors of diffractive layers and potential misalignments induced by imperfect stages/holders can be incorporated as random noise into the physical forward model during the training, making the D^2^NN system resilient to these errors and effectively enhancing robustness in their experimental implementation. Finally, while the discussed D^2^NN applications process input object information under spatially coherent illumination light, recent advancements revealed that D^2^NNs could also function as spatially incoherent optical processing modules^218^. This will open up new possibilities for the use of D^2^NNs in imaging applications employing spatially incoherent terahertz sources.

## Conclusion and future directions

High-throughput terahertz imaging systems will continue to evolve via advancements in both imaging hardware and computational imaging algorithms, targeting faster imagining systems with larger space-bandwidth product, higher sensitivity, and larger dynamic range, while tailoring imaging functionalities for specific applications.

For room-temperature applications where intensity contrast is needed to differentiate between object features, microbolometers and FET image sensors will continue to serve as the key players due to their high sensitivity and scalability. Further developments in the microbolometer and FET image sensor technologies involve the optimization of the terahertz antenna^219^ and integration with metamaterials to improve the terahertz absorption efficiency^220^, as well as scaling to larger arrays while maintaining high imaging speeds. Further sensitivity enhancement could enable room-temperature passive imaging^221^, eliminating the need for a terahertz illumination source and greatly reducing the system complexity and cost. While VO_x_ and α-Si are widely used in room-temperature microbolometers, alternative materials could provide new functionalities in future thermal image sensors. For example, the photothermoelectric effect in 2D materials could be used as the terahertz detection mechanism in future flexible and wearable terahertz image sensor arrays^222^. In terms of scalability, FET image sensors, especially Si metal–oxide–semiconductor field-effect transistor (MOSFET) sensors, would be highly desirable for two reasons: the availability of CMOS foundry processes and the possibility of monolithic integration with the readout electronics. Therefore, FET image sensors are expected to prevail in applications where cost-efficiency matters the most. While 16.4 k-pixel CMOS image sensors with a 73 dB dynamic range have already been demonstrated^35^, larger-pixel CMOS image sensors are anticipated to be available in the near future. Apart from direct terahertz detection, FET image sensors can also be used to perform on-chip heterodyne terahertz detection using integrated phase-locked local oscillators and backend IF electronics. While previously demonstrated heterodyne image sensors had a limited number of pixels^62^, larger-format FET heterodyne image sensors are expected to be realized thanks to the dimensional and functional scaling of CMOS. With large-format image sensors, many snapshot spectroscopic imaging techniques developed for visible and infrared regimes will be feasible for multi-spectral terahertz imaging, such as integral field spectroscopy with lenslet arrays^223^, tunable echelle imaging^224^, and image mapping spectrometry^225^. Many terahertz far-field microscopy techniques, such as dark-field microscopy^226^ and polarized light microscopy^227^, will also benefit from the advanced terahertz sensor arrays.

For intensity imaging applications requiring superior sensitivity, such as astrophysics studies, superconducting image sensors are expected to dominate photon image sensors, at least in the near future. Many challenges remain to be addressed for photon imagers, including challenges in monolithic integration of large-pixel photon image sensor arrays based on quantum wells, quantum dots, and Ge:Ga detectors;^228,229^ challenges in the development of cold readout electronics with sufficiently low thermal emission;^39^ and the limitation of existing extrinsic semiconductors in supporting photon detection at the lower terahertz frequency range^37^. Superconducting image sensors, on the other hand, have demonstrated remarkable sensitivity and scalability, and are the main workhorses in many observatories for astrophysics research^230^. In-depth studies on our universe demand larger-pixel imagers with higher sensitivities. For example, to trace our cosmic history, the Origins Space Telescope requires terahertz image sensors with ~10^4^ pixels and NEP levels as low as 3 × 10^−^^21 ^W/√Hz (sensitivities as high as 3.3 × 10^20^√Hz/W)^47^. TES, KID, and QCD image sensors are all promising candidates for future astrophysics sensing. Among them, TES is the most mature technology. However, further developments are needed to address the fabrication and data readout challenges of large-array TES image sensors. KID image sensors are less mature compared to their TES counterparts but progressed rapidly in recent years. Their highly efficient frequency-domain readout promises great scalability; 961-pixel image sensors are already demonstrated with NEP levels as low as 3 × 10^−^^19 ^W/√Hz (sensitivities as high as 3.3 × 10^18^√Hz/W)^47^. QCD image sensors exhibit superior sensitivity (sensitivities up to 10^20^ √Hz/W, or NEP levels as low as 10^−^^20 ^W/√Hz), while supporting frequency-domain readout^231^. However, they are a less mature technology, and many challenges need to be addressed to increase their fabrication yield, reduce their dark current, and increase their dynamic range.

Photoconductive and electro-optic image sensors will continue to be the pursuit of the future THz-TDS imaging systems enabling time-of-flight, spectroscopic, intensity, and phase imaging simultaneously. Developing high-throughput, large-pixel-count photoconductive image sensor arrays requires new photoconductive terahertz detection schemes that can maintain high SNR and large bandwidth when configured in a large array format. For example, using plasmonic nanoantennas integrated with 3D plasmonic electrodes^232,233^ or plasmonic nanocavities^234–236^ can significantly enhance the terahertz detection sensitivity, while dramatically reducing the required optical probe power while operating at different optical probe wavelengths^237,238^. As a result, high-SNR and broadband operation can be maintained for each pixel, even at a low optical probe budget for a large image sensor array^235,236^. With the development of short-carrier-lifetime, high-mobility, photo-absorbing semiconductors at ~1550 nm wavelength^239,240^, the realization of telecommunication-compatible photoconductive image sensor arrays integrated with femtosecond fiber lasers is another direction that could significantly reduce the cost, size, and complexity of future THz-TDS systems. To achieve faster imaging speeds, advanced 2D readout integrated circuits as well as non-mechanical time-domain sampling methods (e.g., ASOPS and ECOPS) can be used in conjunction with the photoconductive image sensor arrays. For THz-TDS imaging systems based on electrooptic image sensor arrays, expanding the field of view would decrease the terahertz field intensity at each pixel. Therefore, increasing the energy of the terahertz pulses illuminating the imaged object and using nonlinear crystals with higher nonlinearity effects are possible ways to maintain an acceptable SNR and detection bandwidth. Large-format THz-TDS image sensors would bridge the gap between many exciting potentials and real-world applications of terahertz waves to reconstruct 3D images of multi-layered objects and identify chemical composition of unknown objects in real-time, for various industrial quality control, security screening, and health monitoring applications.

Future terahertz imaging systems are also expected to benefit from rapid advancements in computational and data sciences. Multi-pixel sensor arrays utilized with quantum sensing and compressive sensing algorithms^160^ are expected to push the envelope and offer unprecedented functionalities for future terahertz imaging systems. By delving deeper into innovations in 3D fabrication and lithography techniques, particularly using nonlinear materials, the capabilities of terahertz imaging techniques powered by D^2^NNs can further expand, allowing a broader range of applications, including terahertz microscopy, non-line-of-sight imaging and wireless communication. While advancements in terahertz imaging techniques covering both hardware and computational methods will be crucial for future terahertz systems, the interaction of terahertz electromagnetic waves with real-world objects and materials should be carefully studied and modeled to further extend the boundaries of terahertz imaging and sensing applications. We foresee a flourishing future for terahertz imaging science and technology and a significant growth in the utilization of terahertz imaging systems not only in scientific laboratories and industrial settings, but also in our daily lives.

### Supplementary information

Supplementary Information for High-throughput terahertz imaging: progress and challenges
