# Supplementary material for: High-throughput terahertz imaging: progress and challenges
Source: Light Sci Appl. 2023 Sep 15;12:233. doi: 10.1038/s41377-023-01278-0 (PMC10504281; doi:10.1038/s41377-023-01278-0)
Supplement: Supplementary file 1 — Supplementary Information for High-throughput terahertz imaging: progress and challenges [file 41377_2023_1278_MOESM1_ESM.docx]

Supplementary Information for

# High-throughput terahertz imaging: progress and challenges

**Authors:** Xurong Li^1,2^, Jingxi Li^1,2,3^, Yuhang Li^1,2,3^, Aydogan Ozcan^1,2,3^ and Mona Jarrahi^1,2,*^

**Affiliations:**

^1^Department of Electrical & Computer Engineering, University of California Los Angeles (UCLA), California 90095, USA

^2^California NanoSystems Institute (CNSI), University of California Los Angeles (UCLA), California 90095, USA

^3^Department of Bioengineering, University of California Los Angeles (UCLA), California 90095, USA

*Corresponding author. Email: mjarrahi@ucla.edu

| **Ref.** | **Type** | **Operation temperature** | **Pixel count** | **Pixel pitch** | **Frequency range (THz)** | **Frame rate (fps)** | **Noise equivalent power (W Hz^-0.5^)** |
| --- | --- | --- | --- | --- | --- | --- | --- |
| [S1] | Microbolometer image sensors | RT | 110592 | 35 μm | 0.1 - 4.25 | 50 | 0.11×10^-12^ @ 4.25 THz 0.18×10^-12^ @ 2.52 THz 0.18×10^-12^ @ 1.89 THz 0.12×10^-12^ @ 0.762 THz 0.12×10^-12^ @ 0.693 THz 0.31×10^-12^ @ 0.397 THz 0.32×10^-12^ @ 0.198 THz |
| [S2] |  | RT | 1310720 | 12/15 μm | - | 10, 30, 60 | < 1.5×10^-12^ @ 4.6 THz |
| [S3] |  | CT | 512 | 162.56 mm | 1.43 - 2.31 | - | 2.1×10^-16^ |
| [S3] |  | CT | 2048 | 81.28 mm | 2.31 - 5 | - | 2.1×10^-16^ |
| [S4] |  | CT | 2500 | - | 0.65 | 1 | 16×10^-15^ |
| [S5] |  | CT | 8712 | - | 0.4 | 9 | 14×10^-15^ |
| [S6] | Pyroelectric image sensors | RT | 76800 | 80 μm | 0.1 - 10 | 100 | 13×10^-9^ |
| [S7] | Field-effect transistor image sensors | RT | 4096 | 1.5 mm | 0.05 - 0.7 | 50 | 1×10^-9^ |
| [S8] |  | RT | 1024 | 80 μm | 0.1 - 4 | 500 | 100×10^-12^ @ 0.86 THz, 5 kHz chopping |
| [S9] |  | RT | 1024 | 80 μm | 0.46 - 0.75 | 25 | 262×10^-12^ @ 0.653 THz, 40 kHz chopping |
| [S10] |  | RT | 1024 | 215 μm | 0.93 | 400 | 91×10^-12^ @ 0.93 THz, 31 Hz chopping 13.7 ×10^-12^ @ 0.93 THz, 100 kHz chopping |
| [S11] |  | RT | 961 | 240 μm | 0.2 - 0.6 | 25-100 | 18.7×10^-12^ @ 0.27 THz 25.9×10^-12^ @ 0.6 THz |
| [S12] |  | RT | 576 | 195 μm | 0.6 | 450 | 43×10^-12^ @ 0.6 THz |
| [S3] | Photon image sensors | CT | 400 | - | 1.30 - 2.31 | - | 8.9×10^-18^ |
| [S3] |  | CT | 400 | - | 2.31 - 3.75 | - | 2.1×10^-17^ |
| [S13] |  | CT | 1024 | - | 4.3 | - | - |
| [S13] |  | CT | 40 | - | 1.9 | - | - |
| [S14] |  | CT | 16384 | - | 7.5 - 10 | - | - |
| [S15] | Superconducting image sensors | CT | 961 | 1.3 mm | 0.77 - 0.97 | - | 3×10^-19^ |
| [S16] |  | CT | 989 | - | 1.4 - 2.8 | - | 2.8×10^-19^ |
| [S17] |  | CT | 152 | - | 0.35 | 2 | - |
| [S18] |  | CT | 5120 | - | 0.35 | 180 | 5.6×10^-17^ |
| [S18] |  | CT | 5120 | - | 0.67 | 180 | 2.7×10^-16^ |
| [S19] |  | CT | 144 | - | 0.15 | 1 | 2.3×10^-16^ |
| [S19] |  | CT | 256 | - | 0.22 | 1 | 1.5×10^-15^ |
| [S20] |  | CT | 441 | - | 1.5 | 1 | 5×10^-20^ |

**Table S1 | Comparison of frequency-domain terahertz image sensors.** RT: room temperature. CT: cryogenic temperature.

**Reference**

[S1] M. Terroux, P. Talbot, F. Généreux, L. Marchese, E.-H. Oulachgar, and A. Bergeron, “NEP characterization and analysis method for THz imaging devices,” in *Passive and Active Millimeter-Wave Imaging XXIV*, D. A. Robertson and D. A. Wikner, Eds., Online Only, United States: SPIE, Apr. 2021, p. 19. doi: 10.1117/12.2586094.

[S2] “RIGI Camera, Swiss Terahertz.” https://www.swissterahertz.com/rigicamera (accessed Apr. 17, 2023).

[S3] A. Poglitsch *et al.*, “The Photodetector Array Camera and Spectrometer (PACS) on the Herschel Space Observatory,” *A&A*, vol. 518, p. L2, Jul. 2010, doi: 10.1051/0004-6361/201014535.

[S4] A. Timofeev *et al.*, “Optical and Electrical Characterization of a Large Kinetic Inductance Bolometer Focal Plane Array,” *IEEE Trans. THz Sci. Technol.*, vol. 7, no. 2, pp. 218–224, Mar. 2017, doi: 10.1109/TTHZ.2016.2639470.

[S5] J. Luomahaara *et al.*, “A Passive, Fully Staring THz Video Camera Based on Kinetic Inductance Bolometer Arrays,” *IEEE Trans. THz Sci. Technol.*, vol. 11, no. 1, pp. 101–108, Jan. 2021, doi: 10.1109/TTHZ.2020.3029949.

[S6] “Pyrocam IV, Ophir.” https://www.ophiropt.com/laser--measurement/beam-profilers/products/Beam-Profiling/Camera-Profiling-with-BeamGage/Pyrocam-IV (accessed Apr. 17, 2023).

[S7] “Tera-4096, TeraSense.” https://terasense.com/products/sub-thz-imaging-cameras/ (accessed Apr. 23, 2023).

[S8] “TicMOS-1kpx, TicWave.” https://ticwave.com/index.php/shop/ticmos-1kpx (accessed Apr. 23, 2023).

[S9] R. Jain *et al.*, “34.3 A 32×32 Pixel 0.46-to-0.75THz Light-Field Camera SoC in 0.13μ m CMOS,” in *2021 IEEE International Solid- State Circuits Conference (ISSCC)*, San Francisco, CA, USA: IEEE, Feb. 2021, pp. 484–486. doi: 10.1109/ISSCC42613.2021.9365832.

[S10] S. Yokoyama *et al.*, “5.8 A 32×32-Pixel 0.9THz Imager with Pixel-Parallel 12b VCO-Based ADC in 0.18μm CMOS,” in *2019 IEEE International Solid- State Circuits Conference - (ISSCC)*, San Francisco, CA, USA: IEEE, Feb. 2019, pp. 108–110. doi: 10.1109/ISSCC.2019.8662483.

[S11] A. Boukhayma, A. Dupret, J.-P. Rostaing, and C. Enz, “A Low-Noise CMOS THz Imager Based on Source Modulation and an In-Pixel High-Q Passive Switched-Capacitor N-Path Filter,” *Sensors*, vol. 16, no. 3, p. 325, Mar. 2016, doi: 10.3390/s16030325.

[S12] J. Zdanevičius *et al.*, “Camera for High-Speed THz Imaging,” *J Infrared Milli Terahz Waves*, vol. 36, no. 10, pp. 986–997, Oct. 2015, doi: 10.1007/s10762-015-0169-1.

[S13] G. H. Rieke *et al.*, “The Multiband Imaging Photometer for Spitzer (MIPS),” *ASTROPHYS J SUPPL S*, vol. 154, no. 1, pp. 25–29, Sep. 2004, doi: 10.1086/422717.

[S14] Martin C.E. Huber, *Observing Photons in Space: A Guide to Experimental Space Astronomy*, Second Edition. Springer, 2013.

[S15] J. J. A. Baselmans *et al.*, “A kilo-pixel imaging system for future space based far-infrared observatories using microwave kinetic inductance detectors,” *A&A*, vol. 601, p. A89, May 2017, doi: 10.1051/0004-6361/201629653.

[S16] J. Bueno, V. Murugesan, K. Karatsu, D. J. Thoen, and J. J. A. Baselmans, “Ultrasensitive Kilo-Pixel Imaging Array of Photon Noise-Limited Kinetic Inductance Detectors Over an Octave of Bandwidth for THz Astronomy,” *J Low Temp Phys*, vol. 193, no. 3–4, pp. 96–102, Nov. 2018, doi: 10.1007/s10909-018-1962-8.

[S17] S. Rowe *et al.*, “A passive terahertz video camera based on lumped element kinetic inductance detectors,” *Rev. Sci. Instrum.*, vol. 87, no. 3, p. 033105, Mar. 2016, doi: 10.1063/1.4941661.

[S18] W. S. Holland *et al.*, “SCUBA-2: the 10000 pixel bolometer camera on the James Clerk Maxwell Telescope,” *Monthly Notices of the Royal Astronomical Society*, vol. 430, no. 4, pp. 2513–2533, Apr. 2013, doi: 10.1093/mnras/sts612.

[S19] A. Monfardini *et al.*, “A DUAL-BAND MILLIMETER-WAVE KINETIC INDUCTANCE CAMERA FOR THE IRAM 30 m TELESCOPE,” *ApJS*, vol. 194, no. 2, p. 24, Jun. 2011, doi: 10.1088/0067-0049/194/2/24.

[S20] P. M. Echternach, S. van Berkel, A. D. Beyer, G. Chattopadhyay, and C. M. Bradford, “Large Array of Single-Photon Counting Quantum Capacitance Detectors,” *IEEE Trans. THz Sci. Technol.*, vol. 12, no. 2, pp. 211–216, Mar. 2022, doi: 10.1109/TTHZ.2021.3126542.
